# Spectrum and Frequency of the *GJB2* Gene Pathogenic Variants in a Large Cohort of Patients with Hearing Impairment Living in a Subarctic Region of Russia (the Sakha Republic)

**DOI:** 10.1371/journal.pone.0156300

**Published:** 2016-05-25

**Authors:** Nikolay A. Barashkov, Vera G. Pshennikova, Olga L. Posukh, Fedor M. Teryutin, Aisen V. Solovyev, Leonid A. Klarov, Georgii P. Romanov, Nyurgun N. Gotovtsev, Andrey A. Kozhevnikov, Elena V. Kirillina, Oksana G. Sidorova, Lena M. Vasilyevа, Elvira E. Fedotova, Igor V. Morozov, Alexander A. Bondar, Natalya A. Solovyevа, Sardana K. Kononova, Adyum M. Rafailov, Nikolay N. Sazonov, Anatoliy N. Alekseev, Mikhail I. Tomsky, Lilya U. Dzhemileva, Elza K. Khusnutdinova, Sardana A. Fedorova

**Affiliations:** 1 Department of Molecular Genetics, Federal State Budgetary Scientific Institution “Yakut Science Centre of Complex Medical Problems,” Yakutsk, Russian Federation; 2 Laboratory of Molecular Biology, Institute of Natural Sciences, M.K. Ammosov North-Eastern Federal University, Yakutsk, Russian Federation; 3 Laboratory of Human Molecular Genetics, Federal Research Center, Institute of Cytology and Genetics, Siberian Branch of the Russian Academy of Sciences, Novosibirsk, Russian Federation; 4 Novosibirsk State University, Novosibirsk, Russian Federation; 5 Department of Radiology, Republican Hospital # 2 –Center of Emergency Medicine, Ministry of Public Health of the Sakha Republic, Yakutsk, Russian Federation; 6 Republican Centre of Professional Pathology, Republican Hospital # 2 –Center of Emergency Medicine, Ministry of Public Health of the Sakha Republic, Yakutsk, Russian Federation; 7 Institute of Foreign Philology and Regional Studies, M.K. Ammosov North-Eastern Federal University, Yakutsk, Russian Federation; 8 Audiology-Logopaedic Centre, Republican Hospital #1– National Medical Centre, Ministry of Public Health of the Sakha Republic, Yakutsk, Russian Federation; 9 SB RAS Genomics Core Facility, Institute of Chemical Biology and Fundamental Medicine, Siberian Branch of the Russian Academy of Sciences, Novosibirsk, Russian Federation; 10 Department of Biochemistry and Biotechnology, Institute of Natural Sciences, M.K. Ammosov North-Eastern Federal University, Yakutsk, Russian Federation; 11 Institute of Humanitarian Research and Indigenous Peoples of the North, Siberian Branch of the Russian Academy of Sciences, Yakutsk, Russian Federation; 12 Laboratory of Human Molecular Genetics, Institute of Biochemistry and Genetics, Ufa Scientific Centre, Russian Academy of Sciences, Ufa, Russian Federation; 13 Department of Immunology and Human Reproductive Health, Bashkir State Medical University, Ufa, Russian Federation; 14 Department of Genetics and Fundamental Medicine, Bashkir State University, Ufa, Russian Federation; Lyon Neuroscience Research Center, FRANCE

## Abstract

Pathogenic variants in the *GJB2* gene, encoding connexin 26, are known to be a major cause of hearing impairment (HI). More than 300 allelic variants have been identified in the *GJB2* gene. Spectrum and allelic frequencies of the *GJB2* gene vary significantly among different ethnic groups worldwide. Until now, the spectrum and frequency of the pathogenic variants in exon 1, exon 2 and the flanking intronic regions of the *GJB2* gene have not been described thoroughly in the Sakha Republic (Yakutia), which is located in a subarctic region in Russia. The complete sequencing of the non-coding and coding regions of the *GJB2* gene was performed in 393 patients with HI (Yakuts—296, Russians—51, mixed and other ethnicities—46) and in 187 normal hearing individuals of Yakut (n = 107) and Russian (n = 80) populations. In the total sample (n = 580), we revealed 12 allelic variants of the *GJB2* gene, 8 of which were recessive pathogenic variants. Ten genotypes with biallelic recessive pathogenic variants in the *GJB2* gene (in a homozygous or a compound heterozygous state) were found in 192 out of 393 patients (48.85%). We found that the most frequent *GJB2* pathogenic variant in the Yakut patients was c.-23+1G>A (51.82%) and that the second most frequent was c.109G>A (2.37%), followed by c.35delG (1.64%). Pathogenic variants с.35delG (22.34%), c.-23+1G>A (5.31%), and c.313_326del14 (2.12%) were found to be the most frequent among the Russian patients. The carrier frequencies of the c.-23+1G>A and с.109G>A pathogenic variants in the Yakut control group were 10.20% and 2.80%, respectively. The carrier frequencies of с.35delG and c.101T>C were identical (2.5%) in the Russian control group. We found that the contribution of the *GJB2* gene pathogenic variants in HI in the population of the Sakha Republic (48.85%) was the highest among all of the previously studied regions of Asia. We suggest that extensive accumulation of the c.-23+1G>A pathogenic variant in the indigenous Yakut population (92.20% of all mutant chromosomes in patients) and an extremely high (10.20%) carrier frequency in the control group may indicate a possible selective advantage for the c.-23+1G>A carriers living in subarctic climate.

## Introduction

Pathogenic variants in the *GJB2* gene (gap junction protein beta 2, 13q12.11) encoding connexin 26 (Cx26) are known to be a major cause of congenital hearing impairment (HI) in many countries [1]. To date, more than 300 different allelic variants (The Human Gene Mutation Database) have been described in the *GJB2* gene [2]. Spectrum and allelic frequencies of the *GJB2* gene vary significantly among different ethnic groups worldwide [3, 4]. Currently, the regions of Europe [5–32], Asia [33–48], the Middle East [49–56], Central and North America [57–61], South America [62–69], Greenland [70], Australia [71], and some parts of Africa [72–81] have been characterized according to the pathogenic variant spectrum and frequency of the *GJB2* gene. However, data regarding the molecular basis of HI in populations of Russia are scarce [7, 16, 82].

Preliminary mutational analysis of the coding region (exon 2) of the *GJB2* gene in patients with HI from the Sakha Republic (Yakutia) located in subarctic region of Russia (Northeast Asia) revealed the presence of the *GJB2* pathogenic variants in 50.1% of patients of Caucasian origin (Russians, Ukrainians, and Ingushes) and only in 7.2% of the Yakut patients (indigenous population of the Sakha Republic) [83]. Subsequent mutational analysis of the non-coding region of the *GJB2* gene revealed a large cohort of Yakut patients with HI who were homozygous for the splice site pathogenic variant c.-23+1G>A (70 unrelated patients in total) [84]. Nevertheless, until now, the spectrum and frequency of all pathogenic variants in exon 1, exon 2 and the flanking intronic regions of the *GJB2* gene in the Sakha Republic have not been described thoroughly.

In this study, we present updated data on the spectrum and frequency of the *GJB2* gene sequence variants (exon 1, exon 2 and flanking intronic regions) in the extended cohort of patients with HI (n = 393) of different ethnicities and in normal hearing individuals (n = 187) living in the Sakha Republic.

## Materials and Methods

### Patients

Data on individuals with HI were obtained from the Republican Hospital # 1 of the National Medical Centre (Yakutsk, Russian Federation) and the Republican special residential schools for the deaf and hard-of-hearing children (Yakutsk, Russian Federation). The genomic DNA samples of 393 patients with HI from 360 unrelated families were collected from 2005 to 2010. The majority of patients were Yakuts (75.3%; n = 296), Russian patients (12.9%; n = 51), and patients of mixed and other ethnicities (11.7%; n = 46) (Table 1). Audiograms of patients demonstrated variability in bilateral sensorineural HI (from mild to profound). In most cases, the hearing thresholds were determined by pure-tone audiometry, using a clinical tonal audiometer GSI 60 (Grason-Stadler, Madison, WI, USA) in a soundproof room according to the current clinical standards. Air-conduction thresholds were obtained at 0.125, 0.25, 0.5, 1, 2, 4, and 8 kHz. Severity of hearing loss was defined as mild (25–40 dB), moderate (41–70 dB), severe (71–90 dB) or profound (above 90 dB).

**Table 1:** Characteristics of the patients and control groups.

Ethnicity	Patients groups				Control groups			
	Total n = 393	Male	Female	Mean age	n = 187	Male	Female	Mean age
Yakuts	n = 296 (75.3%)	50.3%	49.7%	17.2±1.0 years	n = 107 (57.2%)	31.8%	68.2%	23.7±2.3 years
Russians	n = 51 (12.9%)	58.8%	41.2%	19.0±2.6 years	n = 80 (42.8%)	Data not available	Data not available	Data not available
Individuals of mixed and other ethnicities	n = 46 (11.7%)	56.5%	43.5%	17.6±2.5 years	-	-	-	-

### Control group

The control group was represented by 187 unrelated normal hearing individuals of Yakut (n = 107) and Russian (n = 80) ethnicities living in different districts of the Sakha Republic (Table 1). Blood samples were collected after written informed consent. The carrier frequency of the major *GJB2* pathogenic variants c.-23+1G>A and c.35delG in Yakuts was calculated by a compilation of corresponding data from previous studies [85, 86]. Differences in the c.-23+1G>A pathogenic variant frequencies between the study groups (95% credible interval) were computed with the ‘Sampling’ software kindly provided by V. Macaulay and adapted by M. Metspalu (Estonian Biocentre, Tartu, Estonia).

### Sequence analysis of the *GJB2* gene

DNA was extracted from the blood leukocyte fraction using the phenol-chloroform method. Amplification of non-coding (exon 1), coding (exon 2) and flanking intronic regions of the *GJB2* gene was conducted with PCR on a MJ Mini (Bio-Rad) thermocycler using primers 5'-CCGGGAAGCTCTGAGGAC-3' and 5'-GCAACCGCTCTGGGTCTC-3' for amplification of exon 1 [55] and 5'-TCGGCCCCAGTGGTACAG-3' and 5'-CTGGGCAATGCGTTAAACTGG-3' for amplification of exon 2 [32, 58, 59]. The PCR products were subjected to direct sequencing using the same primers on ABI PRISM 3130XL (Applied Biosystems, USA) Genomics Core Facility, Institute of Chemical Biology and Fundamental Medicine, Siberian Branch of the Russian Academy of Sciences, Novosibirsk, Russia). DNA sequences variations were identified through comparison with the *GJB2* gene reference sequences М86849.2 and U43932.1 (GenBank).

### Epidemiological data

The Sakha Republic (Yakutia), which includes 34 districts and two cities, is the largest (by territory) administrative region of the Russian Federation located in Eastern Siberia with the area of 3103.2 km^2^. The data on population and ethnic composition of each district and city were obtained from the Department of the Federal Service of National Statistics in the Sakha Republic (Yakutia). The total population of the Sakha Republic is 958,528 people (64.1%—urban population), with a density of 0.31 people per km^2^. The major ethnic groups are the Yakuts (48.6%) and the Russians (36.9%). The minor ethnic groups are the Ukrainians (2.1%), the Evenks (2.1%), and the Evens (1.5%). Other ethnic groups are <1%. DNA samples were collected from the patients with HI living in 30 different districts of the Sakha Republic. The prevalence of HI caused by biallelic recessive *GJB2* pathogenic variants was counted per 10,000 people in the Sakha Republic.

### Ethical approval

All written informed consent forms signed by the participants or the guardians of the underage participants involved in our study were obtained before the testing procedures. This study was approved by the local Biomedical Ethics Committee of Federal State Budgetary Scientific Institution “Yakut Science Centre of Complex Medical Problems”, Yakutsk, Russia (Protocol No. 16, April 16, 2009).

## Results

### Spectrum of the *GJB2* gene sequence variants

**Fig 1:**
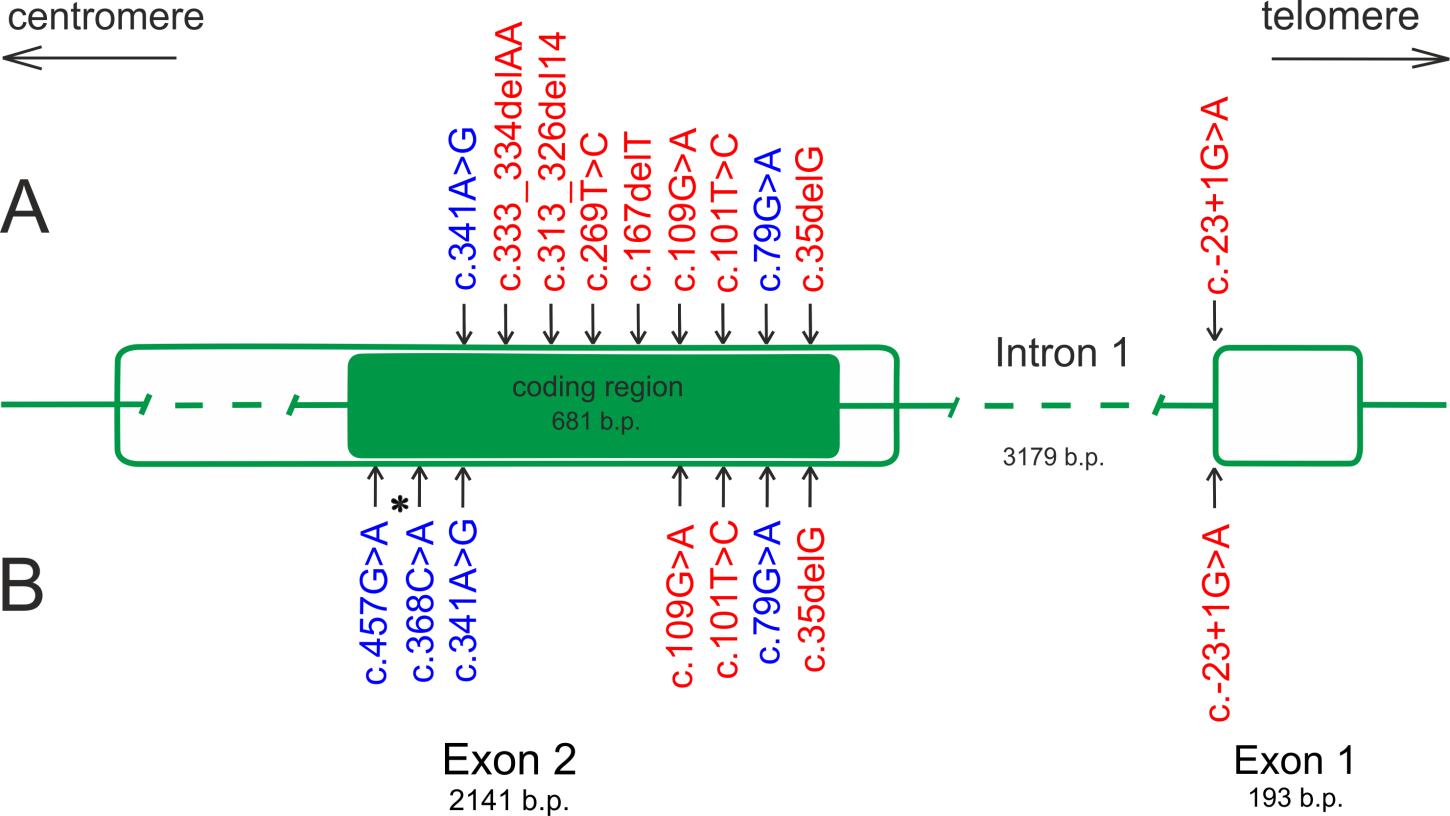
Detected pathogenic and benign variants in the *GJB2* gene in patients (A) and control groups (B). Note: Pathogenic variants are shown in red, benign variants are shown in blue; *—unclassified variant, b.p.—base pair.

Sequencing of the coding (exon 2), non-coding (exon 1) and flanking intronic regions of the *GJB2* gene in 393 patients and 187 controls revealed 12 allelic variants (c.-23+1G>A, c.35delG, c.79G>A, c.101T>C, c.109G>A, c.167delT, c.269T>C, c.313_326del14, c.333_334delAA, c.341A>G, c.368C>A, and c.457G>A) (Fig 1). Among them, eight recessive pathogenic variants associated with HI (c.-23+1G>A, c.35delG, c.101T>C, c.109G>A, с.167delT, c.269T>C, с.313_326del14, and c.333_334delAA), three benign variants (c.79G>A, с.341A*>*G, and с.457G>A), and one unclassified variant (с.368С>A) were detected (Fig 1).

### *GJB2* genotypes in patients

Twenty one different *GJB2* genotypes were identified in all patients with HI (n = 393). Among them, ten pathogenic (biallelic recessive pathogenic variants in a homozygous or compound heterozygous state) *GJB2* genotypes were found in 192 patients (48.85%). Four common pathogenic *GJB2* genotypes were presented with a frequency >1%: с.[-23+1G>A];[-23+1G>A] (37.91%), c.[-23+1G>A];[35delG] (4.58%), c.[35delG];[35delG] (3.56%), and c.[109G>A];[109G>A] (1.01%) (Table 2).

**Table 2:** *GJB2* genotypes in patients with HI.

*GJB2* genotypes		Yakut patients		Russian patients		Patients of mixed and other ethnicities		Total	
Nucleotide level (NM_004004.5)	Amino acid level (NP_003995.2)	n = 296	Frequency (%)	n = 51	Frequency (%)	n = 46	Frequency (%)	n = 393	Frequency (%)
c.[-23+1G>A];[-23+1G>A]	[Splice site, m.RNA];[Splice site, m.RNA]	142	47.97	2	3.92	5	10.86	149	37.91
c.[-23+1G>A];[35delG]	[Splice site, m.RNA];p.[Gly12ValfsX2]	8	2.70	2	3.92	8	17.39	18	4.58
c.[-23+1G>A];[109G>A]	[Splice site, m.RNA];р.[Val37Ile]	2	0.67	-	-	-	-	2	0.50
c.[-23+1G>A];[167delТ]	[Splice site, m.RNA];р.[Leu56ArgfsX26]	-	-	1	1.96	-	-	1	0.25
c.[-23+1G>A];[313_326del14]	[Splice site, m.RNA];p.[Lys105GlyfsX5]	-	-	1	1.96	-	-	1	0.25
c.[-23+1G>A];[333_334delAA]	[Splice site, m.RNA];р.[Ile111IlefsХ2]	-	-	1	1.96	-	-	1	0.25
c.[35delG];[35delG]	p.[Gly12ValfsX2];[Gly12ValfsX2]	-	-	9	17.64	5	10.86	14	3.56
c.[35delG];[109G>A]	p.[Gly12ValfsX2];[Val37Ile]	1	0.33	-	-	-	-	1	0.25
c.[35delG];[313_326del14]	p.[Gly12ValfsX2]; [Lys105GlyfsX5]	-	-	1	1.96	-	-	1	0.25
c.[109G>A];[109G>A]	p.[Val37Ile];[Val37Ile]	4	1.35	-	-	-	-	4	1.01
*GJB2* genotypes with biallelic recessive pathogenic variants in total		157	53.04	17	33.33	18	39.13	192	48.85
c.[-23+1G>A];[wt]	[Splice site, m.RNA];[wt]	17	5.74	-	-	1	2.17	18	4.58
c.[-23+1G>A];[79G>A]	[Splice site, m.RNA];р.[Val27Ile]	5	1.68	-	-	1	2.17	6	1.52
c.[-23+1G>A];[79G>A(;)341A>G]	[Splice site, m.RNA];р.[Val27Ile(;)Glu114Gly]	4	1.35	-	-	-	-	4	1.01
c.[35delG];[wt]	p.[Gly12ValfsX2];[wt]	-	-	2	2.92	1	2.17	3	0.76
c.[101Т>С];[wt]	p.[Met34Thr];[wt]	1	0.33	2	2.92	-	-	3	0.76
c.[109G>A];[wt]	p.[Val37Ile];[wt]	2	0.67	1	1.96	-	-	3	0.76
c.[79G>A];[269Т>С]	p.[Val27Ile];[Ile90Pro]	1	0.33	-	-	-	-	1	0.25
*GJB2* genotypes with single recessive pathogenic variants in total		30	10.13	5	9.80	3	6.52	38	9.66
c.[79G>A];[wt]	p.[Val27Ile];[wt]	21	7.09	1	1.96	5	10.86	27	6.87		
c.[79G>A];[79G>A]	p.[Val27Ile];[Val27Ile]	4	1.35	-	-	1	2.17	5	1.27
c.[79G>A];[79G>A(;)341A>G]	p.[Val27Ile];[Val27Ile(;)Glu114Gly]	1	0.33	-	-	-	-	1	0.25
c.[79G>A(;)341A>G];[wt]	p.[Val27Ile(;)Glu114Gly];[wt]	4	1.35	1	1.96	-	-	5	1.27
*GJB2* genotypes with benign variants in total		30	10.13	2	2.92	6	13.04	40	10.17
*GJB2* genotype [wt];[wt]		79	26.68	27	52.94	19	41.30	125	31.80

Seven different *GJB2* genotypes with single recessive pathogenic variants and wild type allele or benign variants were found in 38 (9.66%) patients. Four different *GJB2* genotypes with benign and unclassified variants were detected in 40 (10.17%) patients. No changes in the *GJB2* gene sequence were found in 125 (31.80%) patients (Table 2).

### Spectrum and contribution of the *GJB2* gene pathogenic variants in HI in two different ethnic groups of patients

**Fig 2:**
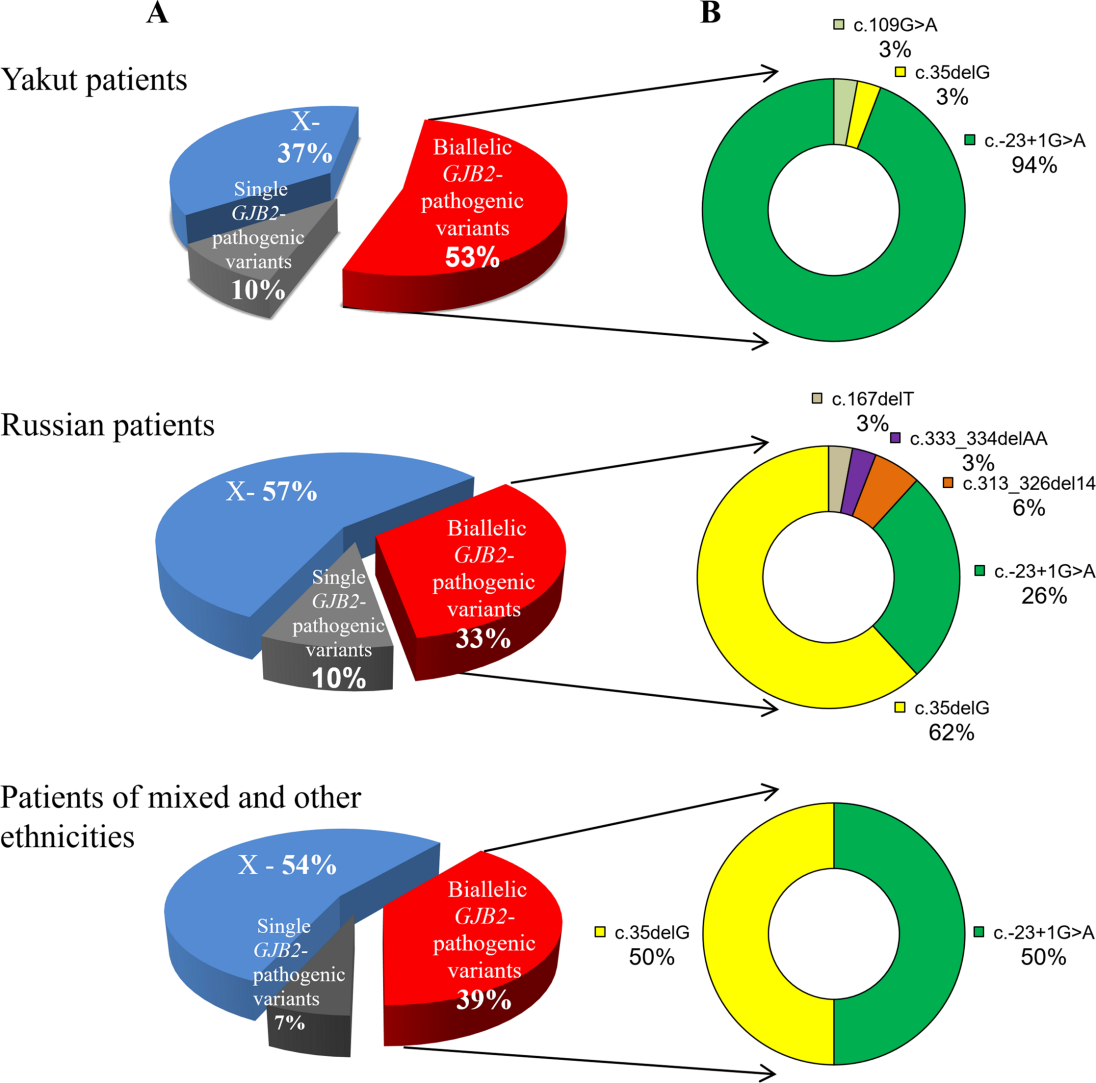
Contribution to HI (A) and spectrum (B) of the pathogenic *GJB2* gene variants among the ethnic groups of patients with HI in the Sakha Republic. Note: Х—no changes were found in the *GJB2* gene sequence.

We found different contributions of the *GJB2* gene pathogenic variants in HI among two major different ethnic groups of patients (Yakuts and Russians). The presence of biallelic recessive *GJB2* pathogenic variants were detected in 157 out of 296 Yakut patients with HI (53.04%) (Table 2). Pathogenic variant c.-23+1G>A was the most frequent (93.63% of all mutant chromosomes) among the three recessive *GJB2* pathogenic variants detected in Yakut patients (Fig 2). The HI in 17 out of 51 Russian patients (33.33%) was caused by the presence of biallelic recessive *GJB2* pathogenic variants. Particularly, c.35delG was the most frequent (61.76% of all mutant chromosomes) among the five pathogenic variants found in the Russian patients (c.-23+1G>A, c.35delG, с.313_326del14, c.333_334delAA, and с.167delT) (Fig 2). Three pathogenic *GJB2* genotypes accounted for HI in 39.13% of patients of the other ethnicities (Fig 2).

### *GJB2* allelic frequencies in patients and control groups

We found that three pathogenic variants, c.-23+1G>A (42.28%), с.35delG (5.92%), and c.109G>A (1.92%), were common (with an allelic frequency >1%) (Table 3).

**Table 3:** Allele frequency of pathogenic and benign *GJB2* variants in patients and control groups.

Allelic variants of the *GJB2* gene		Yakut patients n = 274		Control group (Yakuts) n = 107		Russian patients n = 47		Control group (Russians) n = 80		Patients of mixed and other ethnicities n = 42	
Nucleotide level (NM_004004.5)	Amino acid level (NP_003995.2)	548 chromosomes	АF (%)	214 chromosomes	АF (%)	94 chromosomes	АF (%)	160 chromosomes	АF (%)	84 chromosomes	АF (%)
c.-23+1G>A	Splice site, m.RNA	284	51.82	12	5.60	5	5.31	-	-	18	21.42
c.35delG	p.Gly12ValfsX2	9	1.64	-	-	21	22.34	2	1.25	13	15.47
c.101Т>С	p.Met34Thr	1	0.18	1	0.46	1	1.06	2	1.25	-	-
c.109G>A	p.Val37Ile	13	2.37	3	1.40	1	1.06	-	-	-	-
c.167delТ	p.Leu56ArgfsX26	-	-	-	-	1	1.06	-	-	-	-
c.269Т>С	p.Ile90Pro	1	0.18	-	-	-	-	-	-	-	-
с.313_326del14	p.Lys105GlyfsX5	-	-	-	-	2	2.12	-	-	-	-
c.333_334delAА	p.Ile111IlefХ2	-	-	-	-	1	1.06	-	-	-	-
c.79G>A	p.Val27Ile	34	6.20	7	3.27	1	1.06	-	-	8	9.52
c.[79G>A(;)341A>G]*	p.[Val27Ile(;)Glu114Gly]*	9	1.64	5	2.33	1	1.06	-	-	-	-
c.[79G>A(;)368С>A]*	p.[Val27Ile(;)Thr123Asn]*	-	-	3	1.40	-	-	-	-	-	-
c.457G>A	p.Val153Ile	-	-	-	-	-	-	1	0.62	-	-

Note: allelic frequencies of the *GJB2* pathogenic variants were calculated for unrelated patients; n—number of individuals; АF—allelic frequency; wt—wild type

*—likely *in cis* configuration.

Pathogenic variant c.-23+1G>A was the most frequent (51.82%) among the Yakut patients, followed by c.109G>A (2.37%) and c.35delG (1.64%). In the Yakut control group with high allelic frequency, we found pathogenic variants c.-23+1G>A (5.60%) and c.109G>A (1.40%) and benign variants c.79G>A with c.341A>G (2.33%) and without c.341A>G (3.27%) (Table 2). The c.79G>A in a homozygous state with с.368С>A was found in one individual from the Yakut control group. This finding confirms the *cis*-configuration of two pairs of benign variants (c.79G>A with c.341A*>*G and c.79G>A with 368С>A) reported earlier in studies in Asian populations [33–48, 61]. In Russian patients, the pathogenic variants с.35delG (22.34%), c.-23+1G>A (5.31%) and c.313_326del14 (2.12%) were found to be the most frequent. In the Russian control group, we found two pathogenic variants, c.35delG and c.101T>C, with an identical allelic frequency of 1.25%. Benign variant c.457G>A was found in one individual from the Russian control group (0.62%) (Table 3). Only two pathogenic variants, c.-23+1G>A (21.42%) and с.35delG (15.47%), were found in patients from other ethnic groups (Table 3).

### Carrier frequency of the *GJB2* pathogenic variants in the Yakut and Russian control groups

We estimated the carrier frequency of *GJB2* recessive pathogenic variants in the studied control groups. In the Yakut controls, carrier frequencies of c.-23+1G>A, c.35delG, c.101T>C, and с.109G>A were 10.2%, 0.4%, 0.9% and 2.8%, respectively (Table 4). The carrier frequency of с.35delG and c.101T>C detected in the Russian controls was found to be identical (2.5%) (Table 4).

**Table 4:** Carrier frequency of the major pathogenic variants of the *GJB2* gene in the Yakut and Russian control samples.

Pathogenic variants of *GJB2* gene		Yakuts			Russians		
Nucleotide level (NM_004004.5)	Amino acid level (NP_003995.2)	n = 107	CF	CR	n = 80	CF	CR
c.-23+1G>A	Splice site, m.RNA	36/350*	0.102	0.075–0.139	0/80	-	-
c.35delG	p.Gly12ValfsX2	1/247*	0.004	0.001–0.022	2/80	0.025	0.008–0.086
c.101Т>С	p.Met34Thr	1/107	0.009	0.002–0.051	2/80	0.025	0.008–0.086
c.109G>A	p.Val37Ile	3/107	0.028	0.01–0.079	0/80	-	-

Note: n—number of individuals; CF—carrier frequency; CR—95% credible region.

*—Data on the carrier frequency of pathogenic variants c.-23+1G>A and c.35delG in the Yakut population were compiled from previous studies [85, 86].

### Distribution of HI caused by the presence of biallelic *GJB2* recessive pathogenic variants in the Sakha Republic

**Fig 3:**
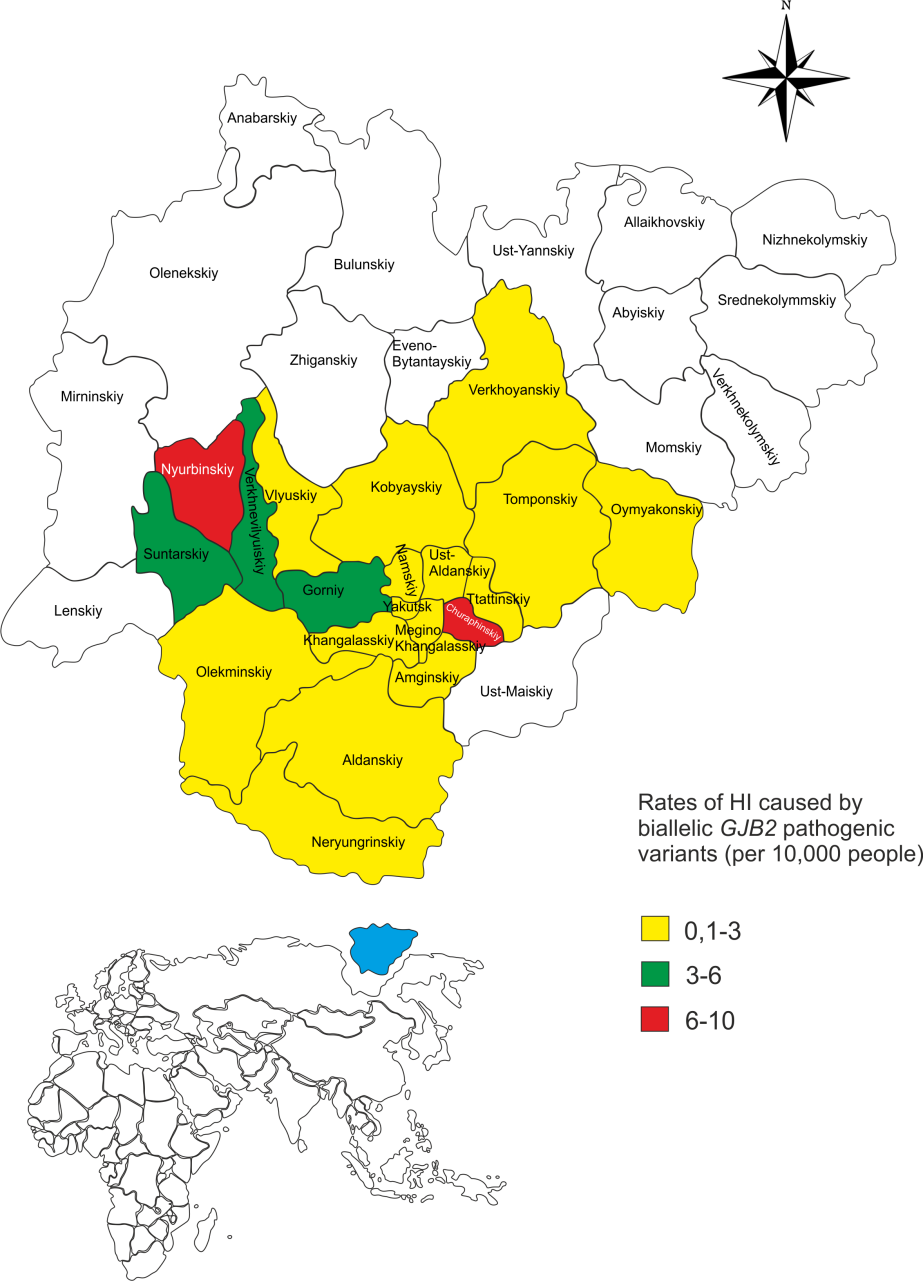
Prevalence of congenital HI caused by biallelic *GJB2* pathogenic variants in the Sakha Republic. Note: The territory of the Sakha Republic is shown in blue (bottom map). HI rates were calculated per 10,000 people, and appropriate data are presented only for the districts and cities of the Sakha Republic with population more than 10,000. Detailed data are presented in S1 Table.

We analyzed the distribution of the *GJB2* genotypes with biallelic recessive pathogenic variants in the Sakha Republic (S1 Table and Fig 3). The average rate of HI caused by the biallelic *GJB2* pathogenic variants was 2.00±0.14 per 10,000, with the highest prevalence in the Nyurbinskiy (9.50±1.94) and Churapchinskiy (7.84±1.96) districts of the Sakha Republic.

## Discussion

In this study, we present updated data on the spectrum and frequency of the *GJB2* gene allelic variants (exon 1, exon 2 and flanking intronic region) in a large cohort of patients with HI (n = 393) and in normal hearing individuals (n = 187) living in the Sakha Republic (in total, n = 580). The majority of patients with HI were Yakuts (indigenous population of the Sakha Republic) and Russians, with the minority being from other ethnic groups (Ukrainians, Evenks, Evens, and Tatars) and individuals of mixed ethnicity.

In total, in the studied samples (n = 580), we revealed 12 allelic variants of the *GJB2* gene, eight of which were recessive pathogenic variants. All identified sequence variants were found in the coding region (exon 2) of *GJB2*, except for the splice site pathogenic variant c.-23+1G>А, which is located in the non-coding region (intron 1) of *GJB2* (Fig 1). The c.-23+1G>A (42.28%), с.35delG (5.92%), and c.109G>A (1.92%) pathogenic variants were found to be common (>1%) in the total patient samples.

Nevertheless, the frequencies of the *GJB2* pathogenic variants in the Yakut and Russian ethnic groups of patients differed significantly. We found that the c.-23+1G>A pathogenic variant was predominant (51.82%) in the Yakut patients with the second most frequent pathogenic variant c.109G>A (2.37%), followed by c.35delG (1.64%). These findings confirm our earlier reported data on the high prevalence of the c.-23+1G>A pathogenic variant among the Yakut population in Eastern Siberia [84]. Interestingly, in the Yakut patients, the second most common pathogenic variant was c.109G>A (p.Val37Ile), which was found with high frequency in Southeast Asia (Thailand, Indonesia, Malaysia) [41, 47, 48], East Asia (China, Korea, Japan) [33, 35–38, 40, 42, 44, 46], and Australia [71], as well as among patients with HI of Asian origin in the US [87]. Pathogenic variants с.35delG (22.34%), c.-23+1G>A (5.31%), and c.313_326del14 (2.12%) were the most frequent in the group of Russian patients. These findings are comparable with earlier reported data on the prevalence of c.35delG, c.-23+1G>A, and c.313_326del14 pathogenic variants among some countries of Eastern Europe: the European region of Russia (30.0%, 1.91%, 2.12%), Czech Republic (35.6%, 2.88%, 1.60%), Slovakia (22.30%, 0.54%, 0.91%) and Croatia (35.30%, 0.90%, 1.70%) [7, 20, 25–27]. Thus, among deaf patients in the Sakha Republic, we found not only a major c.-23+1G>A pathogenic variant but also Asian-specific (c.109G>A) [33–48, 84] and Caucasian-specific (c.35delG) pathogenic variants [5–32, 49–60]. These findings are in accordance with the ethnic composition of the Sakha Republic population (Yakuts—48.6%, Russians—36.9% and other ethnic groups—14.5%).

Data on the territorial distribution of HI caused by genetic factors are of great importance for the clinical evaluation of deaf people and for estimating recurrence risks for their families. In this study, we estimated the distribution of the *GJB2* genotypes with biallelic recessive pathogenic variants in the Sakha Republic (average rate was found to be 2.00±0.14 per 10,000) (S1 Table). The highest prevalence of HI, caused by biallelic *GJB2* recessive pathogenic variants, was registered in the Nyurbinskiy (9.50±1.94) and Churapchinskiy (7.84±1.96) districts of the Sakha Republic (Fig 3). These findings are comparable with our recent data on the extensive accumulation of the c.-23+1G>A splice site pathogenic variant in the *GJB2* gene as a result of the founder effect [84]. Reconstruction of 140 haplotypes with c.-23+1G>A demonstrated that the most recombined haplotypes (more ancient) were found in the same districts (Nyurbinskiy and Churapchinskiy) of the Sakha Republic [84]. The age of c.-23+1G>A in the Yakut population was estimated at approximately 800 years [84]. A more ancient age of the common *GJB2* pathogenic variants was shown for c.35delG in the Caucasian populations (approximately 10000 years) [88], c.235delC in East Asian populations (approximately 11500 years) [89], and p.Trp24* in India (approximately 8800 years) [43].

In total, from all of the samples, we identified 10 pathogenic genotypes (with biallelic recessive *GJB2* pathogenic variants) in 192 out of 393 studied patients (48.85%). Thus, 48.85% of HI in patients in the Sakha Republic could be caused by the *GJB2* pathogenic variants. Previous reports suggested that the contribution of the *GJB2* pathogenic variants in HI in Asian populations was lower than in Europe and the US. The low contribution of the *GJB2* pathogenic variants in HI was demonstrated in Mongolia (4.5%) [45], Japan (7.52%) [46], Thailand (8.4%) [47] and Korea (8.2%) [42]. A higher contribution of the *GJB2* pathogenic variants in HI was found in China (14.9%) [35], Iran (16.1%) [50] and India (21.1%) [39]. Therefore, our results indicate that the contribution of the *GJB2* pathogenic variants to HI (48.85%) in the Sakha Republic located in subarctic part of Russia was the highest among all studied Asian regions.

We estimated that the total carrier frequency of the *GJB2* pathogenic variants in the Yakuts was 0.143 (Table 4). Based on this data, the expected rate of patients with HI (homozygous or compound heterozygous for the *GJB2* gene pathogenic variants) should be approximately 0.005 in the Yakut population (466,492 in total) or approximately 50 per 10,000 people, which is substantially higher than what we found (Fig 3). This bias could be explained by two reasons: first, such theoretical calculations cannot be applied to a relatively small isolated and subdivided Yakut population, and second, there is a possible underestimation of hearing-impaired people due to the known phenotypic variability of HI (from profound to mild) caused by pathogenic variants in *GJB2* gene. In total, 85% of patients demonstrated severe to profound HI, while 14% displayed moderate HI, and 1% displayed mild HI [84].

In contrast, an extremely high prevalence of the c.-23+1G>A pathogenic variant in the indigenous people living in the subarctic region of Russia (up to 13.3% in some sub-populations of the Yakuts) is comparable with the carrier frequency of the HbS allele associated with sickle cell anemia in Africa (a frequency of 10% and higher of the HbS allele was registered only in certain areas of sub-Saharan Africa) [86, 90]. The worldwide carriers’ frequency for different *GJB2* pathogenic variants is very high [3, 85], suggesting a common selective advantage for heterozygous *GJB2* variants on a global scale. The *GJB2* heterozygote advantage might consist of increased resistance to gastrointestinal infections due to the epithelial barrier thickening, as suggested in previous studies [91–96]. We suggest a similar mechanism of heterozygous advantage for the c.-23+1G>A carriers, although further comprehensive studies are needed to elucidate the special features related to the subarctic climate of the Sakha Republic.

## Conclusions

We found that the contribution of the *GJB2* gene pathogenic variants to HI in the population of the Sakha Republic (48.85%) was the highest among all of the regions of Asia studied previously. We suggest that extensive accumulation of the c.-23+1G>A pathogenic variant in the indigenous Yakut population (92.20% of all mutant chromosomes in patients and an extremely high (10.20%) carrier frequency in the control group) may indicate the possible selective advantage of the c.-23+1G>A carriers living in the subarctic climate.

## Supporting Information

S1 Table Distribution of congenital HI caused by biallelic *GJB2* pathogenic variants in administrative units of the Sakha Republic. (DOCX)
