# Supplementary material for: Spectrum and Frequency of the GJB2 Gene Pathogenic Variants in a Large Cohort of Patients with Hearing Impairment Living in a Subarctic Region of Russia (the Sakha Republic)
Source: PLoS One. 2016 May 25;11(5):e0156300. doi: 10.1371/journal.pone.0156300 (PMC4880331; doi:10.1371/journal.pone.0156300)
Supplement: S1 Table — (DOCX) [file pone.0156300.s001.docx]

Supplementary Table

**Distribution of congenital HI caused by biallelic *GJB2* pathogenic variants in administrative units of the Sakha Republic**

| **№** | **Administrative units of the Sakha Republic (districts and cities)** | **Population (on October 2010) *** | **Population density – (per/km^2^)** | **Number of patients with biallelic *GJB2* pathogenic variants** | **Rates of HI caused of biallelic *GJB2*-pathogenic variants (per 10,000)** |
| --- | --- | --- | --- | --- | --- |
| 1 | Yakutsk | 286160 | 79.48 | 52 | 1.81±0.25 (1:5503) |
| 2 | Zhatay | 9504 | 0.30 | 3 | - |
| 3 | Namskiy | 23198 | 1.94 | 5 | 2.15±0.96 (1:4639) |
| 4 | Khangalasskiy | 34052 | 1.37 | 10 | 2.93±0.92 (1:3405) |
| 5 | Megino-Kangalasskiy | 31278 | 2.67 | 6 | 1.91±0.78 (1:5213) |
| 6 | Amginskiy | 17183 | 0.59 | 4 | 2.32±1.16 (1:4295) |
| 7 | Churapchinskiy | 20387 | 1.61 | 16 | 7.84±1.96 (1:1274) |
| 8 | Tattinskiy | 17242 | 0.90 | 3 | 1.73±1.00 (1:5747) |
| 9 | Ust-Aldanskiy | 22155 | 1.21 | 5 | 2.25±1.00 (1:4431) |
| 10 | Gorniy | 11706 | 0.25 | 6 | 5.12±2.09 (1:1951) |
| 11 | Vilyuskiy | 25222 | 0.45 | 7 | 2.77±1.04 (1:3603) |
| 12 | Verkhnevilyuiskiy | 21661 | 0.50 | 13 | 6.00±1.66 (1:1666) |
| 13 | Suntarskiy | 25140 | 0.43 | 12 | 4.77±1.37 (1:2095) |
| 14 | Nyurbinskiy | 25258 | 0.42 | 24 | 9.50±1.94 (1:1052) |
| 15 | Kobyayskiy | 13680 | 0.12 | 2 | 1.46±1.03 (1:6840) |
| 16 | Tomponskiy | 14099 | 0.10 | 1 | 0.70±0.70 (1:14099) |
| 17 | Oimyakonskiy | 10109 | 0.10 | 1 | 0.98±0.98 (1:10109) |
| 18 | Momskiy | 4452 | 0.04 | 1 | - |
| 19 | Verkhnekolymskiy | 4723 | 0.06 | 3 | - |
| 20 | Srednekolymskiy | 7897 | 0.06 | 2 | - |
| 21 | Nizhnekolymskiy | 4664 | 0.05 | - | - |
| 22 | Zhiganskiy | 4296 | 0.03 | 2 | - |
| 23 | Verkhoyanskiy | 12815 | 0.06 | 2 | 1.56±1.06 (1:64075) |
| 24 | Eveno-Bytanayskiy | 2867 | 0.05 | - | - |
| 25 | Olenekskiy | 4127 | 0.01 | 1 | - |
| 26 | Abyiskiy | 4425 | 0.06 | 1 | - |
| 27 | Allaikhovskiy | 3050 | 0.02 | - | - |
| 28 | Ust-Yanskiy | 8056 | 0.06 | - | - |
| 29 | Bulunskiy | 9054 | 0.04 | 1 | 1.10±1.10 (1:9054) |
| 30 | Anabarskiy | 3501 | 0.06 | - | - |
| 31 | Ust-Maiskiy | 8629 | 0.09 | 1 | - |
| 32 | Aldanskiy | 42632 | 0.27 | 2 | 0.46±0.32 (1:21316) |
| 33 | Neryungrinskiy | 82766 | 0.88 | 1 | 0.12±0.12 (1:82766) |
| 34 | Olekminskiy | 26785 | 0.16 | 2 | 0.74±0.52 (1:13392) |
| 35 | Lenskiy | 39765 | 0.51 | 3 | 0.75±0.43 (1:13255) |
| 36 | Mirniy | 75990 | 0.45 | - | - |
|  | Total | 958528 | 0.31 | 192 | 2.00±0.14 (1:4992) |

***Note:*** ** -* Data from the Federal Service of National Statistics in the Sakha Republic (<http://sakha.gks.ru>). Rates of HI causing by biallelic recessive *GJB2*-pathogenic variants are presented for 22 districts and cities with population more than 10,000.
